# Pathogenic avian mycoplasmas show phenotypic differences in their biofilm forming ability compared to non-pathogenic species *in vitro*

**DOI:** 10.1016/j.bioflm.2024.100190

**Published:** 2024-03-06

**Authors:** Salvatore Catania, Marco Bottinelli, Alice Fincato, Annalucia Tondo, Andrea Matucci, Giorgia Nai, Verdiana Righetti, Francesco Abbate, Ana S. Ramírez, Federica Gobbo, Marianna Merenda

**Affiliations:** a Unità Micoplasmi, WOAH Reference Laboratory for Avian Mycoplasmosis (M. Gallisepticum, M. Synoviae), Istituto Zooprofilattico Sperimentale delle Venezie, 37060, Buttapietra, (VR), Italy; b Dipartimento di Scienze Veterinarie, Università di Messina, 98168, Messina, ME, Italy; c Unidad de Epidemiología y Medicina Preventiva, Instituto Universitario de Sanidad Animal y Seguridad Alimentaria (IUSA), Universidad de Las Palmas de Gran Canaria, 35413, Arucas, Spain

**Keywords:** Biofilm, Mycoplasma, Poultry, SEM

## Abstract

Mycoplasmas are known as the minimalist microorganisms in the microbes’ world. Their minimalist nature makes them highly sensitive to the environmental conditions and limits their ability to survive for extended periods outside their animal host. Nevertheless, there are documented instances of mycoplasma transmission over significant distances and this phenomenon may be linked to relatively unexplored abilities of mycoplasmas, such as their capacity to synthesize biofilm—the predominant mode of bacterial growth in nature. The authors decided to establish a method aimed at inducing the clustering of mycoplasma planktonic cells within a biofilm *in vitro* and subsequently assess the capacity of certain avian mycoplasmas to synthesize a biofilm. A total of 299 avian mycoplasma isolates were included in the study, encompassing both pathogenic (*Mycoplasma gallisepticum*, *M. synoviae*, *M. meleagridis*, *M. iowae*) and non-pathogenic species (*M. gallinaceum*, *M. gallinarum*, *M. iners* and *M. pullorum)*. The authors successfully demonstrated the feasibility of inducing avian mycoplasmas to synthetize *in vitro* a biofilm, which can be visually quantified. The only species that did not produce any biofilm was *M. iowae*. In general, the pathogenic mycoplasmas produced greater quantities of biofilm compared to the non-pathogenic ones. Furthermore, it was observed that the ability to produce biofilm appeared to vary, both qualitatively and quantitatively, not only among different species but also among isolates of a single species. Future studies will be necessary to determine whether biofilm production plays a pivotal epidemiological role for the pathogenic avian mycoplasmas.

## Highlights

• The pathogenic avian mycoplasma species produce greater quantities of biofilm compared to the non-pathogenic ones.
• *Mycoplasma iowae* seems to be unable to produce biofilm.
• A standardized laboratory method that allows visual quantification of biofilm was devised.

## 1 Introduction

Mycoplasmas are extremely simple but quite enigmatic bacteria commonly found in many animal species, including humans [1]. Their unique simplicity derives from an extremely small genome, a feature that comes with drawbacks; in fact, these organisms are obliged to a parasitic or saprophytic existence on the host's mucosal surfaces. Indeed, mycoplasmas have limited biosynthetic capabilities and lack a cell wall; this feature makes them also very sensitive to the environmental conditions and, as a consequence, makes them incapable of surviving for extended periods outside their animal host [1]. Despite all this, although appearing inoffensive, many mycoplasma species can cause disease in humans and animals as well. In fact, there are mycoplasma species, such as *Mycoplasma* (*M.*) *gallisepticum* (*Mg*), *M. synoviae* (*Ms*), *M. meleagridis* (*Mm*) and *M. iowae* (*Mi*), that are pathogens of concern in poultry, causing relevant economic losses worldwide. These mycoplasmas are able to infect different host species and can be transmitted both horizontally and vertically [2]. Surprisingly, the implementation of strict biosecurity measures at farm level does not eliminate the risk of the entry of pathogenic mycoplasmas in poultry flocks. Poultry producers have been making significant efforts in order to create and maintain mycoplasma-free breeder stocks with the aim of eliminating the root cause of pathogen dissemination [3]; however, mycoplasma outbreaks keep being detected in the different poultry sectors on a regular basis [4]. This finding indicates that the horizontal transmission of these microorganisms should not be underestimated. It appears that a critical piece of knowledge that could allow us to better understand mycoplasma ecology has, perhaps, been overlooked. In the past, it has been hypothesized that mycoplasmas may have the ability to survive outside their hosts, and the survival time of *Mg* and *Ms* has been studied thoroughly. These species are able to remain viable on different materials, such as rubber, kanekalon, hair, nose, straw, dust, water, feed and egg debris. However, their vitality on these materials lasts for no more than 4 days [5], except for kanekalon and egg debris, on which *Mg* and *Ms* can survive for 9 days and several months, respectively [6,7]. Even though avian mycoplasmas are able to survive in the environment, this circumstance would hardly explain the horizontal transmission of mycoplasmas over long distances. The role of wild birds, in particular house finches, as pathogen spreaders has been investigated, but it appears these animals are not primarily responsible for mycoplasma diffusion among poultry farms. The detection of genetically similar *Mg* isolates in commercial poultry and house finches is reported in the literature [8,9], even though it seems that only one single lineage persisted in the house finches population [9]. Interestingly, it was also reported that the virulence of house finch *Mg* isolates is attenuated in chickens and turkeys [8,10].

The long-distance transmission of mycoplasmas may be attributed to the ability of mycoplasmas to synthesize a biofilm. A biofilm is a community of microorganisms attached to each other and enclosed within a self-produced polymeric matrix, which is in turn adhered to a solid substrate. The biofilm lifestyle has been recognized as the predominant mode of bacterial growth in nature and it has been estimated that 40–80% of all prokaryotes lives within biofilms [11]. The biofilm lifestyle, unlike the planktonic one, is convenient for microorganisms since it protects them from adverse environmental conditions [12] and, at the same time, serves as “diversity incubators” [13]. A certain number of mycoplasma species [14], including *Mg* [15], has been reported of being able to aggregate into a biofilm on both biotic and abiotic surfaces. However, information regarding the possession of this ability by the other mycoplasma species commonly found in the poultry sectors is scarce and limited to only a few strains/species [15,16]. Obtaining new information on the pathogenic mycoplasmas (*Mg*, *Ms*, *Mm* and *Mi*) as well as on the non-pathogenic ones, such as *M. gallinarum*, *M. gallinaceum*, *M. iners* and *M. pullorum*, could reveal new phenotypical differences among these species, potentially allowing to better understanding the puzzling epidemiology of these organisms in the poultry sectors.

Therefore, this research work was designed to achieve the following objectives: 1) identify the optimal method for visualizing the *in vitro*-synthesized biofilm of avian mycoplasmas in a standard microbiology laboratory setting, 2) investigate the biofilm-forming ability of various avian mycoplasma species, and 3) characterize both the macroscopic and microscopic features of the produced biofilm.

## 2 Materials and methods

### 2.1 Mycoplasma isolates

The mycoplasma strains used for determining the optimal method for visualizing the biofilm (preliminary experiments) are listed in Table 1. Two *Mg* strains (*Mg* S6 and *Mg* 6/85), known biofilm producers [15], were used as positive controls; *Mg* strain ts-11 was included in the experiments as it has been reported to be a non-biofilm producer in the literature, as a negative control [15]. In addition, a *Ms* field isolate (15DIA-2516/3f, IZSVe Strain Collection – IZSVe SC) and the *Ms* strain MS-H were included in the preliminary experiments described hereafter.

**Table 1:** Mycoplasma strains used for the preliminary experiments.

Strains	Biofilm forming ability	Note
*Mg* ts-11	No [15]	Vaccine
*Mg* 6/85	Yes [15]	Vaccine
*Mg* S6	Yes [15]	Vaccine
*Ms* MS-H	Unknown	Vaccine
*Ms* strain 15DIA-2516/3f	Unknown	Field isolate

Legend: *Mg* = *M. gallisepticum*; *Ms* = *M. synoviae*. Qt. = Quantity.

For investigating the *in vitro* biofilm-forming ability of various avian mycoplasma species, a total of 299 field mycoplasma isolates were selected from the collection of the Istituto Zooprofilattico Sperimentale delle Venezie (IZSVe). The mycoplasma species included were: *Mg* (105 isolates), *Ms* (105 isolates), *Mm* (15 isolates), *Mi* (15 isolates), *M. gallinaceum* (15 isolates), *M. gallinarum* (15 isolates), *M. iners* (15 isolates) and *M. pullorum* (14 isolates). It was decided to test more than one isolate per species in order to avoid any bias due to intraspecific variability in biofilm formation. The 299 field isolates used in the experiments were collected between 2010 and 2019 during routine diagnostic procedures performed at IZSVe. Isolates collected during a ten-year time frame were included in the study with the aim of increasing the genetic heterogeneity within the study group. The isolates were obtained from both industrial and rural birds (see Table 2).

**Table 2:** Number of the tested viable mycoplasma strains tested categorized by their origin.

*Mycoplasma* species	Industrial chicken	Backyard chicken	Turkey	Others^a^
*M. gallisepticum*	58	12	24	9
*M. synoviae*	63	5	29	1
*M. meleagridis*	0	1	14	0
*M. iowae*	0	0	9	6
*M. gallinaceum*	6	6	0	3
*M. gallinarum*	9	5	0	1
*M. iners*	5	4	0	6
*M. pullorum*	5	3	0	6

a Duck, goose, guinea fowl, pheasant, partridge, quail.

### 2.2 Mycoplasma culture and species identification

The mycoplasma isolates were cultured in separate tubes containing selective culture broth (Avian Mycoplasma Liquid Medium, Mycoplasma Experience, Reigate, UK). The tubes were incubated at 37 ± 1 °C under 5% CO_2_ atmosphere until a colour change and/or turbidity of the medium was observed. *Mycoplasma* species identification, as well as culture purity control, was done carrying out a 16S-rDNA PCR followed by a Denaturing Gradient Gel Electrophoresis (DGGE) using DNA extracted from each culture as described in the literature [17,18].

### *2.3* *Biofilm in vitro production and visualization*

**Fig. 1:**
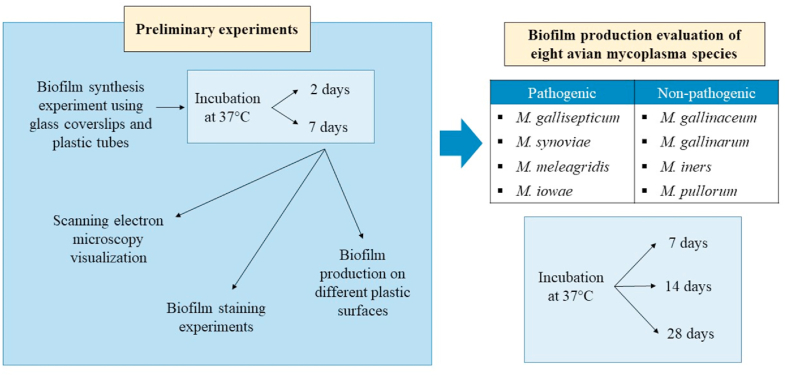
Temporal representation of the experimental steps described in the text.

A graphical flow chart description of the different experiments performed during this project is reported in Fig. 1.

#### 2.3.1 Biofilm synthesis experiments using glass and bijou plastic containers

The procedure chosen to induce mycoplasma planktonic cells to synthetize a biofilm *in vitro* follows the one published by McAuliffe and collaborators [14] with some modifications. The purpose of the preliminary experiments was to assess the ability of a small number of mycoplasma strains (Table 1) to form biofilm on glass and plastic bijou containers.

The ability to form biofilm on glass was evaluated using sterile 22 × 22 mm glass coverslips (Menzel-Gläser, Germany) immersed in 7,5 mL of pre-warmed, sterile culture broth (Avian Mycoplasma Liquid Medium, Mycoplasma Experience, Reigate, UK) contained in polypropylene conical tubes (CLEARLine®, Biosigma, Cona, VE, Italy) – each of 50 mL capacity – with screw-on top closure. Each glass coverslip was placed vertically, at the bottom center of the tube, in a way that only half of it was immerged in the culture broth. A 100 μl culture broth aliquot of each of the strains reported in Table 1 was then glided out on the glass coverslip. For each strain eight glass coverslips were prepared. In addition, a glass in a conical tube was not inoculated and served as negative control. Half of the tubes were then incubated at 37 ± 1 °C under 5% CO_2_ atmosphere for two days and the other half were incubated in the same conditions for seven days. At the end of the incubation period, the glass coverslips were gently washed with a continuous flow of phosphate buffer saline and stained using a crystal violet staining solution at three different concentrations (0,5%, 1% and 2% V/V, two glass coverslips per each stain concentration). After 30 min, the glass coverslips were immerged five times, for 10 min each time, in sterile distilled water in order to remove the excess of staining solution. These were finally left drying up in airtight containers and visually inspected for biofilm detection afterwards. The remaining two out of eight glass coverslips covered with biofilm underwent scanning electron microscopy (SEM) examination.

The ability to form biofilm on plastic was assessed using polystyrene bijou containers with screw-on top closure (Thermo Scientific™ Sterilin™, Thermo Fisher Scientific, Newport, UK). Briefly, eight containers per strain were filled with 2 mL each of culture broth with known titer (1·10^4^ CCU/ml). The containers were then incubated with the same conditions as for the glass coverslips. After the incubation period, the containers were emptied of their content and washed five times with distilled water. The containers underwent to staining procedure using crystal violet staining solutions at three different concentrations (0,5%, 1% and 2% V/V). Two out of eight containers underwent SEM examination.

#### 2.3.2 Biofilm staining experiments

The staining experiments were carried out with the aim of assessing the performances of different staining solutions in showing the biofilm formed by avian mycoplasmas. Plastic bijou containers were chosen on the basis of the results of the previous experiment (see paragraph 2.3.1.). Briefly, 2 mL of culture broth of each strain were poured in a polystyrene bijou container with screw-on top closure (Thermo Scientific™ Sterilin™, Thermo Fisher Scientific, Newport, UK). Four bijou containers were prepared for each isolate and considered as duplicate. One additional container filled with sterile culture broth was used as negative control for each staining experiment. The bijou containers were incubated at 37 ± 1 °C for 7 days. After the incubation period, each bijou container was emptied of its content and rinsed with distilled water. Each of the duplicates underwent a different staining procedure. In Table 3 are summarized the details of the four staining procedures performed. Crystal violet 0,5% (V/V) solution was chosen on the basis of the results of the preliminary experiments (see paragraph 2.3.1.). After the contact time, the bijou containers were rinsed with distilled water and underwent visual staining efficacy assessment.

**Table 3:** List of staining solutions and relative staining procedure (time in contact with biofilm).

Staining solution	Procedure	Producer
Crystal violet 0,5% (V/V)	30 min at room temperature	Sigma-Aldrich
Safranin O 0,25% (V/V)	30 min at room temperature	Sigma-Aldrich
Coomassie Blue	60 min at room temperature	Merck & Co., Inc.
Red Cote	30 min at room temperature	GUM®

#### 2.3.3 Scanning electron microscopy visualization of biofilm

Glass coverslips and the bottoms of the polystyrene bijou containers sent for SEM examination were firstly fixed in 2,5% glutaraldehyde in 0.1 M Sorensen phosphate buffer. After several rinses in the same buffer solution, the different materials were dehydrated with a series of alcohol solutions at increasing concentrations. Then, they were subjected to the critical point dryer procedure by means of a CPD 030 apparatus (BAL-TEC AG, Balzers, Liechtestein), metallized with a 3 nm gold layer in a SCD 050 apparatus (BAL-TEC AG, Balzers, Liechtestein) and examined by means of a EVO LS 10 scanning electron microscope (Zeiss, Oberkochen, Germany) operating at a voltage acceleration of 20 kV.

#### 2.3.4 Biofilm formation experiments using different kinds of plastic plates

The ability of forming biofilm on plastic was further evaluated enrolling different plastic well-plates commonly used in diagnostic laboratories. Three plates of each kind were used for this experiment. For each strain listed in Table 1, four wells were filled with planktonic cell culture and considered as replicates (with the exception of the 6-well microplate). Four additional wells in each plate were filled with sterile culture broth and served as negative control for the session. The volume of the planktonic cell culture poured in each well was different for each plate kind in order to reach the same volume/air ratio. The list of plastic well-plates and the volume of culture broth employed in each experiment is reported in Table 4. After filling the wells with the culture broth and seeding the different mycoplasma isolates, the plates were sealed with a plastic film and incubated at 37 ± 1 °C under 5% CO2 atmosphere for three, seven and fourteen days. At the end of the different incubation periods, the plastic containers were emptied from their content and, after being washed with phosphate buffer saline, underwent to staining procedure with crystal violet 0,5% (V/V) solution.

**Table 4:** **List of plastic plates included in the experiment 2.3.4..** In the middle, the volume of culture broth put into contact with the different surfaces.

Material	Inoculum	Producer
96-well microplate, U-bottom	100 μL	Greiner
96-well microplate for ELISA reader, flat bottom	100 μL	Thermo scientific
96-well microplate TC, flat bottom	100 μL	Costar®, Corning®
48-well microplate TC, flat bottom	500 μL	Falcon®, Corning®
24-well microplate TC, flat bottom	1 mL	Greiner
6-well microplate TC, flat bottom	5 mL	Costar®, Corning®

Legend: TC = Tissue Culture.

#### 2.3.5 Assessment of the biofilm forming ability in the eight mycoplasma species

**Fig. 2:**
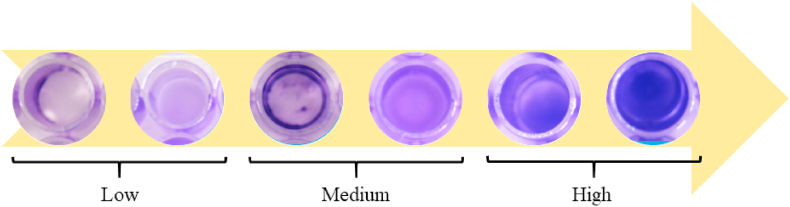
Scoring system used for the visual assessment of the biofilm produced by the *Mycoplasma* isolates in the plastic wells.

On the basis of the results of the preliminary experiments it was chosen to test the 299 mycoplasma field isolates in 48-well plates (Falcon®, Corning®, Durham, USA). Briefly, a 500 μL aliquot of planktonic culture of each isolate was poured in the wells and, after having sealed the well plate with a plastic film, these were incubated at 37 ± 1 °C under 5% CO_2_ atmosphere for 7, 14 and 28 days. The isolates were seeded into three wells each time (intended as replicates) to ensure result consistency. Sterile selective medium for mycoplasma was added to three wells of each plate as a negative control. At the end of the different incubation periods, the wells were emptied from their content, rinsed with phosphate buffer saline and stained with a crystal violet 0,5% (V/V) solution. Then, the well plates were visually inspected; each well was considered as negative (=non-biofilm producer, NBP) or positive (=biofilm producer, BP) for biofilm apposition. As second step, the quantity of biofilm produced was visually assessed and each isolate was scored as low-, medium- or high-biofilm producer according to the scoring system adopted (Fig. 2) at day 28 of incubation. This scoring system takes into consideration the quantity (color intensity) and the distribution of the biofilm formed on the bottom of a well.

## 3 Results

### 3.1 Preliminary experiments using glass and plastic

The biofilm formation experiments carried out using glass coverslips showed that the BP strains were able to produce a visible layer of biofilm, which never crossed the liquid's surface boundary. The BP strains were able to produce a visible biofilm in the polystyrene bijou containers as well. Among the crystal violet staining concentrations tested in these experiments, the 0,5% V/V solution yielded the best results in terms of biofilm colouring. No difference was observed between the days of incubation (2 and 7) in terms of quantity of biofilm produced.

### 3.2 Biofilm staining experiments

The crystal violet 0,5% (V/V) staining solution proved to be the most effective among the staining solutions used for visualization biofilm on plastic (see SM1). Safranin O solution also allowed for the visualization of biofilm, although not for all the strains included in the experiment. The other staining solutions tested were not able to visualize the biofilm in the plastic containers.

### 3.3 Biofilm formation experiments using different kinds of plastic plates

**Fig. 3:**
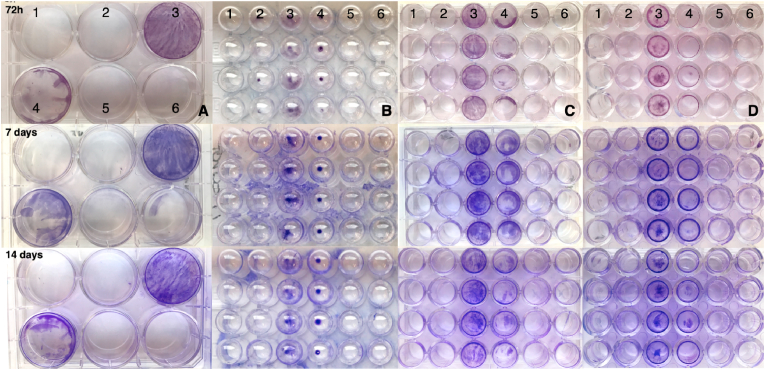
**Visualization of biofilm synthetized on plastic surfaces through crystal violet 0.5% (V/V) staining.** The time of reading is reported on the left for each row. A) 6-well microplate TC-treated, flat bottom; B) 96-well microplate, U-bottom; C) 24-well microplate TC-treated, flat bottom; D) 48-well microplate TC-treated, flat bottom; 1) Negative control; 2) *Mg* ts-11, 3) *Mg* 6/85, 4) *Mg* S6, 5) *Ms* MS-H, 6) *Ms* 15DIA-2516/3f. The negative control as well as each of the strains were inoculated in four wells (columns on the plates).

It was not observed any relevant difference among BP isolates in biofilm formation on the different plastic materials that were used for the experiment (see Fig. 3), except for the ELISA reader plate in which a possible detachment of the biofilm from the bottom of the wells may have occurred (Data not shown). The bottom of the wells in the different kind of plates in which a BP strain had been inoculated was intensely coloured, and it was possible to observe that the biofilm layer was not homogeneous on the whole bottom surface (Fig. 3A–C and D). On the other hand, an intensely coloured dot was observed in the U-bottom wells (Fig. 3B). The authors decided to exclude this kind of plate from the subsequent tests since it did not allow visualizing biofilm distribution of the plastic surface. In addition, it was noted that some mycoplasma strains were able to produce a visible biofilm layer already at 72 h post-incubation. However, it was decided to extend the incubation period to 28 days for the subsequent experiments to allow also slower strains to express their BP-phenotype. The only *Mg* strain that did not produce any visible biofilm during the experiment was Mg ts-11 (Fig. 3-nº2), whereas *Mg* 6/85 (Fig. 3-nº3) and *Mg* S6 (Fig. 3-nº4) strains made a clearly visible biofilm. The tested *Ms* isolates (Fig. 3-nº5 and 6) produced no biofilm.

### 3.4 Scanning electron microscopy visualization of biofilm

**Fig. 4:**
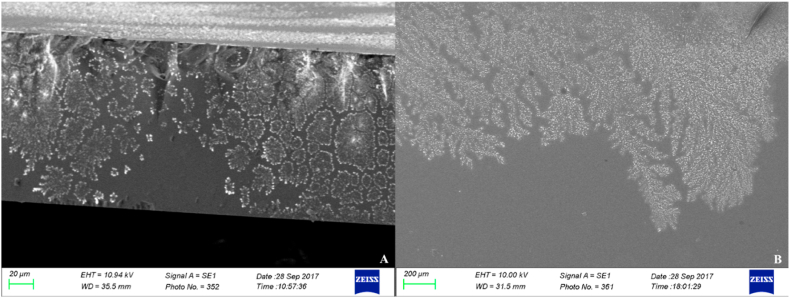
**Scanning electron microscopy examination of glass coverslips derived from the preliminary experiments.** A) MG 6/85 strain incubated for 1 week at 37 °C. B) MS-H strain incubated for 1 week at 37 °C. Irregular, lichen-like shapes can be observed, likely stemming from the detachment of the biofilm from the substrate during the transportation of the glass coverslips to the laboratory.

**Fig. 5:**
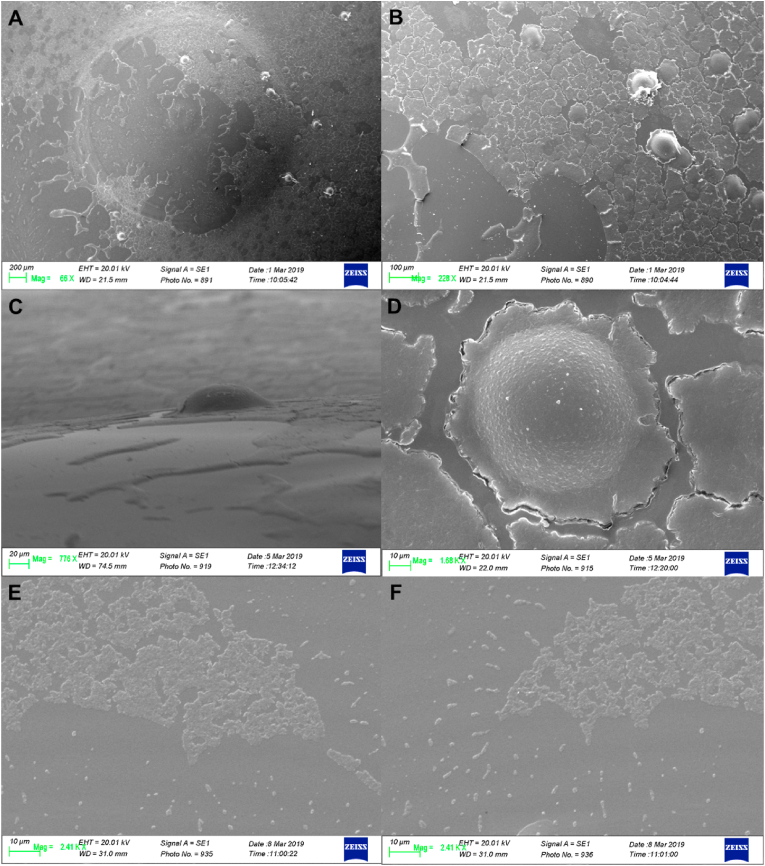
**Scanning electron microscopy examination of the bottom of plastic bijou containers derived from the preliminary experiments.** Incubation time = 7 days. A) and B) *Mg* 6/85 strain, cupoliform formations observed at different magnifications; C) and D) *Mg* S6 strain, cupoliform formations observed from a different angle and at greater magnification so it is possible to appreciate a raised appearance. E) and F) *Mg* ts-11 strain, three-dimensional formations potentially attributable to biofilm observed at two different magnifications.

After SEM examination of the glass coverslips, it was possible to observe the presence of organic structures. When comparing the two incubation periods (two and seven days), distinct microscopic appearances could be observed. In general, organized structures, which can remember the semblances of the classic fried-egg colony of mycoplasmas, were observed. However, lichen-like and ice arabesque structures were found as well, indicating a possible biological growth (lichen-like) occurring on the substrate (see Fig. 4). In addition to these organized structures, numerous simple roundish structures were observed. The SEM examination of the interior bottom of the plastic bijou containers allowed the observation of three-dimensional structures with different strain-related morphology (see Fig. 5). Interestingly, strain *Mg* 6/85 biofilm exhibits cupola- or igloo-shaped structures. Three-dimensional structures resembling potential biofilm primordia were observed for *Mg* ts-11, but these were much less complex compared to those of *Mg* 6/85.

### 3.5 Biofilm formation evaluation

**Fig. 6:**
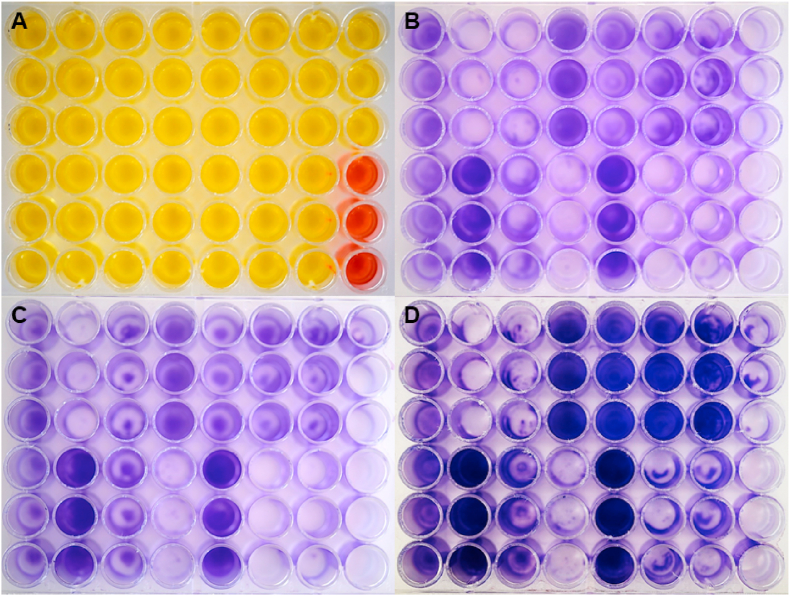
Biofilm formation assessment of different MG strains cultured in 48-well plates. A) Plate containing planktonic culture of *Mycoplasma gallisepticum* isolates (yellow) and sterile culture broth (red). Each isolate was seeded into three vertically consecutive wells. B), C) and D). Plates containing the same isolates as the plate in figure A, and incubated at 7, 14 and 28 days respectively. Cristal violet staining reveals both qualitative and quantative differences in biofilm formation of the different isolates over time.

Out of 299 field mycoplasma isolates, 290 were found to be viable (see Table 5), subjected to purity checks, and assessed *in vitro* for their biofilm forming ability. Since no relevant difference in terms of quantity of biofilm synthetized at day 3 and day 7 was observed during the preliminary experiments, it was decided to stain the plastic plates at 7, 14 and 28 days of incubation. The results of the assessment of the biofilm forming ability of the avian mycoplasma isolates are summarized in Table 5, Table 6. Concordance among the replicates of each strain indicated a high degree of reproducibility of the experiment (see Fig. 6). The species that recorded the highest percentage (96,1%) of isolates capable to form a biofilm was *Mg*, while the one with the lowest number of biofilm forming isolates was *M. pullorum* (14,3%). The only species that was not able, under the reported *in vitro* conditions, to cluster into a biofilm was *Mi*. Crystal violet staining of the biofilm in the plastic wells revealed a high degree of variability in both qualitative and quantitative terms among the different mycoplasma species tested. More than the 85% of *Mg*, *Ms* and *Mm* isolates showed to be able to form a biofilm layer on the bottom of the wells. Most of these isolates, at least for *Mg* and *Mm*, produced a biofilm already after 7 days post-incubation. However, only approximatively half of the BP*-Ms* isolates were able to synthetize a clearly visible biofilm at day 7 post-incubation. In general, a low rate of biofilm forming isolates was observed among the non-pathogenic avian mycoplasma species. The biofilm layer produced by these species was fainter and/or sparse/scattered compared to the one produced by *Mg*, *Ms* and *Mm*. In addition, all the BP-isolates of the non-pathogenic species did not cluster into a biofilm any earlier than 14 days of incubation, except for one isolate of *M. pullorum* (see S1). Most of *Ms* (70,7%) and *Mm* (80,0%) isolates were ranked as low-BP according to the scoring system adopted. Interestingly, the only species of which isolates were ranked as high-BP was *Mg*. Forty-one (41,4%) out of 99 BP-*Mg* isolates were high-BPs. All the biofilm producing isolates belonging to the non-pathogenic avian mycoplasma species were ranked as low-BPs, except for one isolate of *M. gallinaceum* that was ranked as medium-BP.

**Table 5:** **Evaluation of the biofilm produced by avian mycoplasmas over time.** On the left, the list of mycoplasma species included in the experiment. The number of isolates that formed biofilm at different times (7, 14 and 28 days) and the percentage of isolates that formed a biofilm during the experiment is reported in the other columns. Biofilm assessment was conducted using the scoring system created by the authors.

*Mycoplasma* species	7 days	14 days	28 days	Biofilm forming isolates
*M. gallisepticum*	94	97	99	96,1% (99/103)
*M. synoviae*	39	61	84	85,7% (84/98)
*M. meleagridis*	13	13	13	86,7% (13/15)
*M. iowae*	0	0	0	0,0% (0/15)
*M. gallinaceum*	0	5	7	46,7% (7/15)
*M. gallinarum*	0	4	4	26,7% (4/15)
*M. iners*	0	1	7	46,7% (7/15)
*M. pullorum*	1	2	2	14,3% (2/14)

**Table 6:** Results of the biofilm formation evaluation. The percentage of isolates that exhibited or not biofilm production is reported for each of the species included in the study. Visual assessment of biofilm production was conducted at day 28 of incubation.

*Mycoplasma* species	NBP	L	M	H
*M. gallisepticum*	3,88% (4/103)	30,30% (30/99)	28,28% (28/99)	41,41% (41/99)
*M. synoviae*	15,31% (15/98)	83,34% (70/84)	16,67% (14/84)	0%
*M. meleagridis*	13,33% (2/15)	92,31% (12/13)	7,69% (1/13)	0%
*M. iowae*	100% (15/15)	0%	0%	0%
*M. gallinaceum*	53,33% (8/15)	85,71% (6/7)	14,29% (1/7)	0%
*M. gallinarum*	73,33% (11/15)	100,00% (4/4)	0%	0%
*M. iners*	53,33% (8/15)	100,00% (7/7)	0%	0%
*M. pullorum*	85,71% (12/14)	100,00% (2/2)	0%	0%

Legend: NBP = Non-biofilm producer; L = Low biofilm producer; M = Medium biofilm producer; H=High biofilm producer.

## 4 Discussion

The results obtained from the experiments described in this manuscript demonstrate the feasibility of inducing avian mycoplasma planktonic cells to synthetize an *in vitro* biofilm, which can be visually assessed. Additionally, it was observed that the ability to produce biofilm appears to vary, both qualitatively and quantitatively, not only among different species but also among isolates of a single species.

At the initial stages of this research, the authors lacked information about the possible composition of the biofilm produced by avian mycoplasmas. Consequently, they opted to utilize four dyes, namely crystal violet, safranin red, Congo red and red cote, that had been previously employed for assessing the total biomass of biofilms generated by both Gram-positive and Gram-negative bacteria [[19], [20], [21]]. Cristal violet and Safranin red bind to negative charges, thereby revealing various bacterial molecules and extracellular polymeric substances. However, these dyes do not enable the differentiation between viable and dead cells within the biofilm. Despite staining the same biological target, it has been reported that safranin red may be less sensitive than crystal violet in detecting low amounts of biofilm [20]. The authors observed that crystal violet was slightly more effective in evidencing the biofilm, particularly when present in small quantities. Congo red is typically employed for measuring the production of amyloid fibers and determining the quantity of polysaccharides and cellulose within biofilms [22]. Phloxine B, present in GUM® Red-cote dye, binds to positively charged proteins [23] and bacteria [24]. However, in this study, neither Congo red nor Phloxine B demonstrated the ability to stain the biofilm produced by mycoplasmas. In conclusion, crystal violet proved to be the only dye capable to clearly indicate the presence of the biofilm produced by avian mycoplasmas. For this reason, it was selected for subsequent tests in the course of the research.Given that biofilm is produced by bacteria under stressful conditions, the authors chose to adequately and uniformly stress all the mycoplasma strains in order to standardize the experiment as much as possible. Specifically, a static system was employed, eliminating the exchange of nutrients and oxygen over time, resulting in suboptimal growing condition for mycoplasmas. Additionally, the metabolic activity of the tested mycoplasma strains led to a general modification in pH (increase/decrease) in the culture medium, placing additional stress on the planktonic forms in suspension. As shown in Fig. 6, the observable change in color of the culture broth (shifting from orange to deep yellow) and the corresponding variation in pH were evident during the experiment. All the strain tested have altered the pH of the culture medium, indicating that each propagated strain experienced stress during the experiment.

Three out of the four pathogenic avian mycoplasmas have clearly showed their ability to cluster within a biofilm *in vitro*. In fact, more than 90% of *Mg*, and more than 85% *Ms* and *Mm* isolates produced a biofilm after 28 days of incubation. *Mg* was the species whose isolates were most likely to be BPs; in fact, 96.1% of the isolates was able to produce biofilm after 4 weeks of incubation. Furthermore, it is interesting to note that, after just 7 days of incubation, 91.3% (94/103) of *Mg* isolates had already produced a biofilm. Regarding the non-pathogenic avian mycoplasma species tested, a total of 7 *M. gallinaceum* isolates, 4 *M. iners* isolates, 7 *M. gallinarum* isolates and 2 *M. pullorum* isolates synthesized a biofilm after 28 days of incubation. Therefore, we can state that *Mg*, *Ms* and *Mm* are the avian mycoplasma species that are more prone to cluster within a biofilm. In addition, it is noteworthy that none of the non-pathogenic species was able to synthetize biofilm during the first 7 days of incubation, except for a single isolate of *M. pullorum* (isolate 2436/2013). Surprisingly, the only species that did not produce any biofilm under our experimental conditions is *Mi*. Even though the tested isolates were collected during a 10-years time-frame from different bird species with the aim of increasing the heterogeneity of the study group, the authors are aware that these finding will need to be confirmed in the future, reproducing the experiment with a greater number isolates, which will have to come from many different geographical areas. This is particularly true for the *Mi* isolates included in the experiment, since these derive from a limited number of Italian outbreaks [[25], [26], [27]]. Considering that *Mi* is a low-prevalence species in Italy, we cannot exclude that other genetically different isolates may be capable of producing a biofilm. It is also possible that this species may produce a biofilm if placed under different *in vitro* conditions. Otherwise, it is possible that *Mi* is actually unable to produce a biofilm; this characteristic would potentially explain the scarce horizontal transmissibility over long distances observed for this species (data not published yet), which is a well-known vertically transmitted pathogen that appears to survive for no more than 6 days in the environment [5].

Equally interesting is the phenotypical heterogeneity among the biofilms observed during the experiment. Indeed, through electron microscopy images, it was possible to appreciate how the biofilm aggregates can vary outwardly from isolate to isolate (see Fig. 5). The architecture of the biofilm produced by other mycoplasmas has already been studied; *M. pneumoniae* is able to form volcano-like structures [28], while *M. pulmonis* creates tower structures *in vitro* [29]. Images taken with a scanning electron microscope of the biofilm produced by *Mg* during another study [15] showed the presence of lichen-like structures, which are very similar to those created by *M. fermentans* [30] and *Ureaplasma parvum* [31]. In another study on *Ms* [16], both mushroom-like and tower-like structures were observed in the biofilm, even though the authors investigated the biofilm produced as soon as the culture broth was acidified. Interestingly, we found a *Mg* isolate (S6 strain) that was able to produce cupoliform, igloo-like structures on plastic (see Fig. 5), although the biofilm was observed with SEM at fixed times disregarding what was going on in the culture broth. If we attempt to compare our results with those obtained by Chen and collaborators [15], it is possible to observe that the morphology of the biofilm of the Mg 6/85 and ts-11 strains is different. However, the growth conditions (culture medium and incubation time) between the two experiments are different, making it challenging to directly compare the results. Indeed, it is known that the architecture of the biofilm is influenced by various factors, including hydrodynamic conditions and the concentration of available nutrients, and therefore a different biofilm architecture corresponds to a different physiology of the cells living in it. However, this was not the goal of the present study, which aimed at finding a standardized method to efficiently reveal whether a certain mycoplasma isolate is able or not to synthetize a biofilm.

Some heterogeneity was also visually observed after the application of the crystal violet solution in the plastic wells. In particular, the localization, the distribution and the color intensity of the biofilm was variable among the tested isolates. In fact, some isolates produced a thick biofilm evenly distributed on the bottom of the well; differently, other isolates produced a thick biofilm that was located mainly on the inner perimeter of the well. Such variability was seen among the different species but also among the isolates of the same species. In particular, taking *Mg* as an example, it was possible to notice that ten isolates (9,7%) already showed a high production of biofilm after one week of incubation. Other 31 isolates (30,1%) produced the same amount of biofilm only after 4 weeks (see Fig. 6). Compared to the other avian mycoplasma species, *Mg* isolates are the only ones that synthesized a high quantity of biofilm. In addition, the isolates of this species were faster in producing biofilm, which was also thick, in most cases. Lastly, it is worth noting that some *Mg* isolates that were slower in producing a biofilm were classified as low-BPs after seven days of incubation; however, the quantity of biofilm produced increased progressively over time (from the first to the fourth week of incubation) to the point where they could be classified as “slow-producers”.

Quali-quantitative differences were also observed in the other avian mycoplasma species, albeit with less variability. In fact, most of *Ms* isolates turned out being medium- or low-BP. Furthermore, the recorded production rate was lower than the average of the *Mg* isolates; in fact, it was possible to see the formation of a biofilm starting from the second week of incubation. As for *Mg*, the amount of biofilm produced by *Ms* isolates gradually increased over time during the experiment. However, even though *Ms* can be considered, at least *in vitro*, a species capable of producing biofilm, this skill seems to be weaker compared to *Mg*. As regards *Mm*, a different behavior was observed; in fact, albeit in low quantity, the BP-isolates already synthetized a biofilm during the first week, except for one of these. Furthermore, production remained low even after 4 weeks of incubation, with one exception in one isolate that was ranked as medium-BP. As regards the non-pathogenic species, it is interesting to note that only one isolate of *M. iners* synthesized biofilm already at the end of the first week (isolate n.2436/2013), while all the other isolates produced a biofilm after two weeks. However, the isolates of the non-pathogenic species did not appear to be good BPs; in fact, they were all classified as low-BP throughout the experiment, with one exception: an isolate (n.751/2013) of *M. gallinaceum*. Variability in biofilm forming ability among strains of the same species has already been observed for both human [28,32] and animal [14,15,33,34] mycoplasmas. In addition, different variants of the same strain may exhibit variability in terms of gene expression and biological behavior [35]. The strain-related variability observed in the present research work should be looked at in greater depth since it appears that the pathogenic avian mycoplasmas are more likely to be BPs compared to the non-pathogenic species. In fact, this phenotypic feature may be behind the higher pathogenicity and diffusivity of certain isolates.

Over the past two decades, biofilms have been attracting increasing attention in microbiology, ecology and medicine. In fact, the literature contains hundreds of scientific articles about this fascinating form of life shared by many microorganisms [36,37]. A biofilm is a complex entity that shows characteristics of multicellular organisms, a system that grows, metabolizes nutrients, and responds to environmental stimuli [38]. Within the biofilm, microorganisms are able to overcome adverse environmental conditions (e.g., extreme temperature, ultraviolet radiation, high salinity, extreme pH, overcrowding, lack of nutrients) [12], as well as evading significant immune responses mounted by the host, while also evolving at a genetic level [13]. Observing this biological behavior with an evolutionary lens, biofilm could be considered as a full-fledged system of conservation of the species, an outcome of the adaptive divergence to adverse phenomena, resembling other survival mechanisms found in microbiology, such as sporulation for the genera *Bacillus* and *Clostridium* [39,40]. The proof that the biofilm lifestyle is the winning strategy in the “tiny world” is that 40–80% of the prokaryotes lives in biofilms [11]; unfortunately, biofilm communities are also responsible for more than 80% of all chronic infections and constitute a major medical challenge [41]. Therefore, increasing the knowledge about this mode of microbial life becomes fundamental, putting aside for a while the traditional single-cell-centric view of microbiology research. It is reported in the literature that at least fifteen mycoplasma species, of both human [30,32] and animal [14,15,29,33,42] origin, are capable of producing biofilm. The authors demonstrated that five more mycoplasma species are able to produce biofilm *in vitro*, even though the non-pathogenic avian mycoplasmas species showed to be less capable of expressing this phenotype compared to the pathogenic ones.

Considering that mycoplasmas are endowed with this mechanism of protection and conservation, it is legit to reconsider the diffusion dynamics and maintenance of mycoplasmas in animal populations, such as those of the poultry industry. Interestingly, prevalence data in Northern Italy (personal data) would seem to indicate that the transmission of avian mycoplasmas could vary according to the species of *Mycoplasma* considered. In fact, *Mg* and *Ms* are the species mainly isolated −if not the only ones− from industrial poultry, where a depopulation of poultry installation is carried out between animal batches. As a result of this operation, there is no possibility of contact between positive and naïve animals. In rural farms and in game birds [26], where there are animals that live longer, there is promiscuity among different avian species (both domestic and wild) and there is no strict sanitary standard, non-pathogenic mycoplasma species are, on the contrary, more frequently detected. Having found that pathogenic avian mycoplasmas are more likely to produce a biofilm, it is legit speculating on the fact that these species have higher probabilites of surviving in the environment and of being transported over long distances. On the contrary, non-pathogenic avian mycoplasmas probably spread in the animal population mainly by direct contact between infected and healthy animals a common occurrence in rural settings. Their diffusion through fomites, in adverse environmental conditions, could be negligible. However, future studies need to confirm this interesting hypothesis.

Finally, as the connection between biofilm synthesis ability and pathogenicity has not yet been confirmed for mycoplasmas, the question arises as to why not all avian mycoplasmas are capable of forming biofilm clusters, despite this ability being widespread among many microorganisms on Earth [11]. One possible explanation could lie in the process of degenerative evolution that mycoplasmas have undergone over time [43], which potentially led to the loss of genes, including those involved in biofilm formation, from their ancestors. Genes associated with biofilm formation and adhesion have been identified in *Mg* [44]. Biofilm formation has been proposed as a virulence phenotype in both Gram-positive [45,46] and Gram-negative bacteria [47]. Conversely, the ability of *M. bovis* to produce biofilms does not seem to be linked with its pathogenicity [33]. Certainly, future in-depth investigations on this topic are necessary in order to gain a better understanding of the role of biofilms in mycoplasma pathogenicity.

## 5 Conclusions

Although there is some interspecific variability, all of the studied avian mycoplasma species demonstrated the capability of producing biofilms, except for *Mi*. In particular, the pathogenic species such as *Mg*, *Mm* and *Ms*, produced greater quantities of biofilm compared to the non-pathogenic ones. Moreover, the ability to form biofilms appeared to be strain-related. These observations could potentially explain the recurrent outbreaks caused by the same mycoplasma species observed in the poultry industry, as these species manage to survive on abiotic surfaces. Furthermore, this research has facilitated the development of a standardized laboratory system that enables avian mycoplasmas to produce biofilms, which can be visually quantified. Certainly, future large-scale studies will be necessary to gain a better understanding of the differences between the biofilms generated by different *Mycoplasma* species*,* as well as the phenotype expressed in this state of "resistance" in the environment, which probably plays a crucial epidemiological role.

## Confilcts of interest

The authors declare no conflict of interest.

## Funding

Funding for this study was provided by the 10.13039/100009647Italian Ministry of Health (Grant RC IZSVe 13/15 “Micoplasmi aviari e biofilm: un potenziale punto critico per il contenimento di questi patogeni”). The Italian Ministry of Health had no involvment in the preparation of this article, the study design, data collection, analysis interpretation of data, or the decision to submit the article for publication.

## CRediT authorship contribution statement

**Salvatore Catania:** Writing – original draft, Supervision, Project administration, Funding acquisition, Conceptualization. **Marco Bottinelli:** Writing – review & editing, Writing – original draft, Visualization. **Alice Fincato:** Writing – review & editing, Visualization, Investigation. **Annalucia Tondo:** Investigation. **Andrea Matucci:** Investigation. **Giorgia Nai:** Investigation. **Verdiana Righetti:** Investigation. **Francesco Abbate:** Visualization, Investigation. **Ana S. Ramírez:** Writing – review & editing. **Federica Gobbo:** Resources, Investigation, Conceptualization. **Marianna Merenda:** Supervision, Project administration.

## Declaration of competing interest

The authors declare that they have no known competing financial interests or personal relationships that could have appeared to influence the work reported in this paper.

## Data Availability

Data will be made available on request.

## References

[1] Razin S., Hayflick L. (2010). Highlights of mycoplasma research—an historical perspective. Biologicals.

[2] Ferguson‐Noel N., Armour N.K., Noormohammadi A.H., El‐Gazzar M., Bradbury J.M. (2020). Diseases of poultry.

[3] Kleven S.H. (2008). Control of avian mycoplasma infections in commercial poultry. Avian Dis.

[4] Chaidez-Ibarra M.A., Zuleika Velazquez D., Enriquez-Verdugo I., Castro Del Campo N., Angel Rodriguez-Gaxiola M., Montero-Pardo A., Diaz D., Gaxiola S.M. (2021). Pooled molecular occurrence of Mycoplasma gallisepticum and Mycoplasma synoviae in poultry. A systematic review and meta-analysis.

[5] Yavari C.A., Mcbain A.J., Bradbury J.M. (1994). Investigations into the survival of Mycoplasma gallisepticum, Mycoplasma synoviae and Mycoplasma iowae on materials found in the poultry house environment. Avian Pathol.

[6] Chandiramani N.K., Van Roekel H., Olesiuk O.M. (1966). Viability studies with Mycoplasma gallisepticum under different environmental conditions. Poult. Sci..

[7] Abolnik C., Gouws J. (2014). Extended survival times of Mycoplasma gallisepticum and Mycoplasma synoviae on kanekalon synthetic hair fibres. Poult. Sci..

[8] Ferguson N.M., Hermes D., Leiting V.A., Kleven S.H. (2003). Characterization of a naturally occurring infection of a Mycoplasma gallisepticum house finch-like strain in Turkey breeders. Avian Dis.

[9] Hochachka W.M., Dhondt A.A., Dobson A., Hawley D.M., Ley D.H., Lovette I.J. (2013). Multiple host transfers, but only one successful lineage in a continent-spanning emergent pathogen. Proc. R. Soc. B Biol. Sci..

[10] Pflaum K., Tulman E.R., Beaudet J., Liao X., Dhondt K.V., Dhondt A.A., Hawley D.M., Ley D.H., Kerr K.M., Geary S.J. (2017). Attenuated phenotype of a recent house finch-associated Mycoplasma gallisepticum isolate in domestic poultry. Infect Immun.

[11] Flemming H.C., Wuertz S. (2019). Bacteria and archaea on Earth and their abundance in biofilms. Nat Rev Microbiol.

[12] Yin W., Wang Y., Liu L., He J. (2019). Biofilms: the microbial “protective clothing” in extreme environments. Int J Mol Sci.

[13] McDougald D., Rice S.A., Barraud N., Steinberg P.D., Kjelleberg S. (2011). Should we stay or should we go: mechanisms and ecological consequences for biofilm dispersal. Nat Rev Microbiol.

[14] McAuliffe L., Ellis R.J., Miles K., Ayling R.D., Nicholas R.A.J. (2006). Biofilm formation by mycoplasma species and its role in environmental persistence and survival. Microbiology.

[15] Chen H., Yu S., Hu M., Han X., Chen D., Qiu X., Ding C. (2012). Identification of biofilm formation by Mycoplasma gallisepticum. Vet Microbiol.

[16] Kang T., Zhou M., Yan X., Song S., Yuan S., Yang H., Ding H., Jiang H., Zhang D., Bai Y., Zhang N. (2023). Biofilm formation and correlations with drug resistance in Mycoplasma synoviae. Vet Microbiol.

[17] McAuliffe L., Ellis R.J., Lawes J.R., Ayling R.D., Nicholas R.A.J. (2005). 16S rDNA PCR and denaturing gradient gel electrophoresis; a single generic test for detecting and differentiating Mycoplasma species. J Med Microbiol.

[18] Catania S., Gobbo F., Ramirez A.S., Guadagnini D., Baldasso E., Moronato M.L., Nicholas R.A.J. (2016). Laboratory investigations into the origin of Mycoplasma synoviae isolated from a lesser flamingo (Phoeniconaias minor). BMC Vet Res.

[19] Stiefel P., Rosenberg U., Schneider J., Mauerhofer S., Maniura-Weber K., Ren Q. (2016). Is biofilm removal properly assessed? Comparison of different quantification methods in a 96-well plate system. Appl Microbiol Biotechnol.

[20] Ommen P., Zobek N., Meyer R.L. (2017). Quantification of biofilm biomass by staining: non-toxic safranin can replace the popular crystal violet. J Microbiol Methods.

[21] Sahni K, Khashai F, Forghany A, Krasieva T, Wilder-Smith P. (2016). Exploring mechanisms of biofilm removal. Dentistry (Sunnyvale).

[22] Bély M., Makovitzky J. (2006). Sensitivity and specificity of Congo red staining according to Romhányi. Comparison with Puchtler's or Bennhold's methods. Acta Histochem.

[23] Merk (2017). https://www.sigmaaldrich.com/IT/it/product/mm/115926#product-documentation.

[24] Rasooly R. (2007). Phloxine B, a versatile bacterial stain. FEMS Immunol Med Microbiol.

[25] Catania S., Gobbo F., Bilato D., Fincato A., Battanolli G., Iob L. (2012). Isolation of Mycoplasma iowae in commercial Turkey flocks. Vet Rec.

[26] Catania S., Gobbo F., Rodio S., Fincato A., Qualtieri K., Santone C., Nicholas R.A.J. (2014). First isolation of mycoplasma iowae in grey partridge flocks. Avian Dis.

[27] Bottinelli M., Stefani E., Matucci A., Dal Prà M., Capello K., Zotti A., Catania S. (2021). Isolation of Mycoplasma iowae in Turkey flocks with skeletal abnormalities. a retrospective study.

[28] Simmons W.L., Daubenspeck J.M., Osborne J.D., Balish M.F., Waites K.B., Dybvig K. (2013). Type 1 and type 2 strains of Mycoplasma pneumoniae form different biofilms. Microbiology.

[29] Simmons W.L., Dybvig K. (2009). Mycoplasma biofilms ex vivo and in vivo. FEMS Microbiol Lett.

[30] Awadh A.A., Kelly A.F., Forster-Wilkins G., Wertheim D., Giddens R., Gould S.W., Fielder M.D. (2021). Visualisation and biovolume quantification in the characterisation of biofilm formation in Mycoplasma fermentans. Sci Rep.

[31] Rowlands R.S., Kragh K., Sahu S., Maddocks S.E., Bolhuis A., Spiller O.B., Beeton M.L. (2021). A requirement for flow to enable the development of Ureaplasma parvum biofilms in vitro. J Appl Microbiol.

[32] García-Castillo M., Morosini M.I., Gálvez M., Baquero F., del Campo R., Meseguer M.A. (2008). Differences in biofilm development and antibiotic susceptibility among clinical Ureaplasma urealyticum and Ureaplasma parvum isolates. J Antimicrob Chemother.

[33] Bürki S., Frey J., Pilo P. (2015). Virulence, persistence and dissemination of Mycoplasma bovis. Vet Microbiol.

[34] Perez K., Mullen N., Canter J.A., Ley D.H., May M. (2020). Phenotypic diversity in an emerging mycoplasmal disease. Microb Pathog.

[35] Gaurivaud P., Lakhdar L., Le Grand D., Poumarat F., Tardy F. (2014). Comparison of in vivo and in vitro properties of capsulated and noncapsulated variants of Mycoplasma mycoides subsp. mycoides strain Afadé: a potential new insight into the biology of contagious bovine pleuropneumonia. FEMS Microbiol Lett.

[36] Penesyan A., Paulsen I.T., Kjelleberg S., Gillings M.R. (2021). Three faces of biofilms: a microbial lifestyle, a nascent multicellular organism, and an incubator for diversity. NPJ biofilms microbiomes.

[37] Rather M.A., Gupta K., Mandal M. (2021). Microbial biofilm: formation, architecture, antibiotic resistance, and control strategies. Braz J Microbiol.

[38] Rumbaugh K.P., Sauer K. (2020). Biofilm dispersion. Nat Rev Microbiol.

[39] Dürre P. (2014). Physiology and sporulation in Clostridium. Microbiol Spectr.

[40] Stragier P. (2014). A gene odyssey: exploring the genomes of endospore-forming bacteria. Bacillus subtilis Its Closest Relat.

[41] National Institutes of Health (2002). https://grants.nih.gov/grants/guide/pa-files/pa-03-047.html.

[42] Simmons W.L., Bolland J.R., Daubenspeck J.M., Dybvig K. (2007). A stochastic mechanism for biofilm formation by Mycoplasma pulmonis. J Bacteriol.

[43] Himmelreich R., Hubert H., Plagens H., Pirkl E., Li B.C., Herrmann R. (1996). Complete sequence analysis of the genome of the bacterium Mycoplasma pneumoniae. Nucleic Acids Res.

[44] Wang Y., Yi L., Zhang F., Qiu X., Tan L., Yu S., Cheng X., Ding C. (2017). Identification of genes involved in Mycoplasma gallisepticum biofilm formation using mini-Tn4001-SGM transposon mutagenesis. Vet Microbiol.

[45] Cucarella C., Tormo M.Á., Úbeda C., Trotonda M.P., Monzón M., Peris C., Amorena B., Lasa Í., Penadés J.R. (2004). Role of biofilm-associated protein bap in the pathogenesis of bovine Staphylococcus aureus. Infect Immun.

[46] Futo M., Opašić L., Koska S., Corak N., Široki T., Ravikumar V., Thorsell A., Lenuzzi M., Kifer D., Domazet-Lošo M., Vlahoviček K., Mijakovic I., Domazet-Lošo T. (2021). Embryo-like features in developing Bacillus subtilis biofilms. Mol Biol Evol.

[47] Sauer K., Cullen M.C., Rickard A.H., Zeef L.A.H., Davies D.G., Gilbert P. (2004). Characterization of nutrient-induced dispersion in Pseudomonas aeruginosa PAO1 biofilm. J Bacteriol.

